# Preharvest soybean meal supplementation alters tissue-specific protein and amino acid composition in *Morchella sextelata*: integrated transcriptomic and proteomic analysis

**DOI:** 10.3389/fnut.2026.1979592

**Published:** 2026-10-19

**Authors:** Yihong Yue, Qian Wang, Yuchen Zhang, Tingting Xiao, Haibo Hao, Zongjun Tong, Wei Li, Jingjing Huang, Jinjing Zhang, Hui Chen

**Affiliations:** 1 National Engineering Research Center of Edible Fungi, Key Laboratory of Edible Fungi Resources and Utilization (South), Ministry of Agriculture, Institute of Edible Fungi, Shanghai Academy of Agricultural Sciences, Shanghai, China; 2 Amway (China) Botanical Research and Development Center, Wuxi, Jiangsu, China

**Keywords:** exogenous nutrient bags, hydrolyzed amino acids, *Morchella sextelata*, soybean meal supplementation, tissue-specific responses, transcriptome–proteome integration

## Abstract

Preharvest nutrient inputs can modify mushroom composition, but whether graded supplementation differentially affects the protein and amino acid composition of anatomically distinct fruiting-body tissues remains unclear. This study aimed to characterize the compositional and molecular responses of the cap and stipe of *Morchella sextelata* to additional soybean meal. Protein and 18 hydrolyzed amino acids were quantified following 0–10% supplementation, while the control, 2%, 4%, and 8% treatments were analyzed by transcriptomics and proteomics. Protein content responded nonlinearly and was highest at 8% in both tissues; total and essential amino acid contents were also highest in 8% caps. However, cap protein-normalized essential amino acid proportionality did not improve correspondingly, distinguishing compositional enrichment from amino acid balance. Caps generally exhibited more differentially expressed genes, whereas stipes contained more differentially expressed proteins, with downregulated proteins predominating in the 2 and 8% stipe comparisons. Integrated analyses associated these responses with carbon and amino acid metabolism, translation, protein homeostasis, energy metabolism, and RNA processing. These findings demonstrate tissue- and molecular-layer-dependent responses to preharvest nutrient supplementation. Future studies should determine whether the associated pathways translate into altered enzyme activities, metabolic fluxes, and sensory-relevant metabolites.

## 1 Introduction

*Morchella* spp. are high-value edible mushrooms whose commercial value depends on both fruiting-body yield and nutritional composition. Morels contain appreciable amounts of protein together with nutritionally relevant amino acids, polysaccharides, minerals, vitamins, lipids, and other bioactive constituents (1–5). Protein content estimated from total nitrogen reflects the abundance of protein-related nitrogenous constituents, whereas amino acid profiles and protein-normalized amino acid indices provide complementary information on protein composition and amino acid-based nutritional evaluation (6–9). Among cultivated morels, *Morchella sextelata* provides a particularly relevant system for tissue-resolved nutritional investigation. Previous compositional analysis demonstrated that its anatomically differentiated cap and stipe tissues differ substantially in protein, amino acid, carbohydrate, mineral, and bioactive-component contents (10). These established tissue differences make *M. sextelata* suitable for determining whether cultivation-stage nutrient inputs produce distinct compositional responses in different regions of the harvested fruiting body.

Commercial morel cultivation relies on exogenous nutrient bags to sustain nutrient acquisition after soil colonization and support subsequent fruiting-body formation (11, 12). Nutrient-bag formulation is therefore a controllable preharvest factor that may influence the chemical composition of harvested fruiting bodies. Soybean meal is a protein-rich, multicomponent plant material that supplies organic nitrogen, carbohydrates, minerals, and other nutrients that may be utilized during fungal growth and fruiting-body development. It therefore represents more than a source of organic nitrogen alone. Previous substrate-formulation studies have shown that cultivation inputs can affect mushroom growth and composition, although the direction and magnitude of these effects depend on the fungal species and substrate. In *Hericium erinaceus*, graded inclusion of soybean-meal-derived material altered substrate nitrogen content, mycelial growth, and biological efficiency (13). Reformulation with agricultural residues or nutrient-rich ingredients also changed fruiting-body protein, amino acid, and proximate composition in *Pleurotus ostreatus*, *Lentinula edodes*, *Auricularia cornea*, and *Hypsizygus marmoreus* (14–17). The ability of mushroom-forming fungi to convert plant-derived substrates into protein-enriched fungal biomass further indicates that fruiting-body composition depends on both substrate formulation and fungal nutrient-conversion capacity (18). Collectively, these findings demonstrate that substrate composition can influence cultivation performance and harvested fruiting-body composition. However, previous studies primarily compared alternative formulations or emphasized yield and broad proximate composition, leaving the graded and tissue-resolved compositional responses of cultivated morels insufficiently understood.

Evidence linking graded additional soybean meal supplementation in exogenous nutrient bags to the tissue-specific protein and hydrolyzed amino acid composition of morel fruiting bodies remains limited (11, 12, 19). Three questions therefore remain. First, it is unknown whether increasing soybean meal supplementation proportionally increases protein and hydrolyzed amino acid contents. Second, it is unclear whether protein and EAA contents per unit dry weight change consistently with protein-normalized EAA composition. Third, the cap and stipe may exhibit different transcriptomic and proteomic responses at equivalent supplementation levels. Protein-related composition in fungal fruiting bodies is associated with nutrient availability, central carbon and energy metabolism, and downstream processes involving amino acid activation, translation, protein processing, and quality control (20–22). Multi-omics studies of morel mycelial growth have also revealed coordinated changes in carbon, amino acid, and energy metabolism, but did not address responses in mature cap and stipe tissues (23, 24). Substrate reformulation in *Lentinula edodes* altered fruiting-body traits together with molecular responses involving nutrient utilization and amino acid metabolism (25). Tissue-resolved studies have also revealed distinct compositional and molecular profiles between the cap and stipe of *Pleurotus giganteus* and among tissues of *Dictyophora indusiata* (26, 27). However, the tissue- and concentration-dependent compositional, transcriptomic, and proteomic responses of mature *M. sextelata* across graded levels of additional soybean meal remain unclear.

In this study, additional soybean meal was incorporated into the basal exogenous nutrient-bag formulation at 0%, 1%, 2%, 4%, 8%, and 10% of the basal formulation dry weight. Protein content, hydrolyzed amino acid composition, amino acid scores (AAS), and the essential amino acid index (EAAI) were determined separately in the caps and stipes of mature fruiting bodies. RNA sequencing and quantitative proteomics were performed for the control, 2%, 4%, and 8% treatments to characterize molecular responses associated with the observed compositional variation. This study aimed to determine how graded preharvest soybean meal supplementation affects protein content, hydrolyzed amino acid composition, and amino acid-based nutritional indices in the cap and stipe of mature *M. sextelata* fruiting bodies and to characterize the accompanying tissue- and concentration-associated transcriptomic and proteomic responses.

## 2 Materials and methods

### 2.1 Culture conditions and sample preparation

The *M. sextelata* strain Shen Yang No. 4 was maintained at the Institute of Edible Fungi, Shanghai Academy of Agricultural Sciences (Shanghai, China). The cultivation experiment was conducted at a greenhouse production base in Qingpu District, Shanghai, China. Spawn was sown in December 2024, and all treatments were managed using the same conventional field cultivation practices. The basal exogenous nutrient-bag formulation contained 45% wheat, 45% corn cobs, 5% soybean meal, 3% corn flour, 1% lime, and 1% gypsum on a dry-weight basis. The soybean meal used for supplementation contained 7.27% total nitrogen on a dry-weight basis. Additional soybean meal was added at 0% (control, CK), 1%, 2%, 4%, 8%, or 10% relative to the 600-g dry weight of the basal formulation. The bags were sterilized at 121 °C for 2 h and placed on the cultivation soil 1 week after sowing.

Mature fruiting bodies were identified by fully developed reticulate ridges and pits on the pileus, deep and well-expanded pits, a fleshy pileus texture, and firm stipe tissue without the fragility characteristic of immature fruiting bodies. Fifteen mature fruiting bodies were collected from each treatment and divided into three biological replicates, with five fruiting bodies per replicate. Caps and stipes from the five fruiting bodies within each replicate were pooled separately and divided among compositional and molecular analyses. Molecular samples were frozen in liquid nitrogen and stored at −80 °C, whereas compositional samples were dried. For RNA sequencing and proteomics, the control (CK), 2%, 4%, and 8% treatments were designated CK, low (L), medium (M), and high (H), respectively. Cap samples were designated CKC, LC, MC, and HC, whereas stipe samples were designated CKS, LS, MS, and HS. Following compositional analysis, the 2%, 4%, and 8% treatments were selected for transcriptomic and proteomic analyses because they formed a twofold supplementation series representing low, intermediate, and high levels. The 8% treatment also produced the highest measured protein content in both tissues. The 1 and 10% treatments were retained in the compositional analyses to characterize the lower end of the supplementation range and the post-peak response, respectively.

### 2.2 Determination of protein and hydrolyzed amino acid contents

Samples were dried at 80 °C to constant weight, ground, and passed through a 40-mesh sieve. Protein content was estimated from Kjeldahl nitrogen using a conversion factor of 6.25. Approximately 0.3 g of powder was digested with 10 mL of concentrated sulfuric acid, and nitrogen was determined using a K1100 automatic Kjeldahl analyzer (Shandong Hanon Instruments Co., Ltd., Shandong, China). Results were expressed as g kg^−1^ dry weight (DW).

Hydrolyzed amino acids other than tryptophan were determined by acid hydrolysis, precolumn phenyl-isothiocyanate derivatization, and HPLC. Powder (0.2 g) was hydrolyzed with 1 mL of 6 mol L^−1^ HCl containing 0.1% phenol at 100 °C for 20 h. The hydrolysate was dried under nitrogen, reconstituted in 1 mL of 0.1 mol L^−1^ HCl, derivatized using norleucine as the internal standard, extracted with n-hexane, and filtered through a 0.45-μm membrane. Analysis was performed using an L3000 HPLC system (RIGOL Technologies Co., Ltd., Beijing, China) equipped with a Sepax C18 column (250 × 4.6 mm, 5 μm; Sepax Technologies, Inc., Suzhou, China) at 40 °C, with a flow rate of 1.0 mL min^−1^, an injection volume of 10 μL, and detection at 254 nm. Amino acids were quantified against authentic standards with internal-standard correction and expressed as mg g^−1^ DW. Tryptophan was determined by incubating 0.02 g of powder with 1 mL of alkaline hydrolysis reagent at 40 °C for 18 h. After adding 1 mL of water and centrifuging at 10,000 ×*g* for 10 min, the supernatant was analyzed at 590 nm using a commercial kit (Suzhou Comin Biotechnology Co., Ltd., Suzhou, China) and a Synergy H1 microplate reader (BioTek Instruments, Inc., USA). Results were expressed as mg g^−1^ DW. Three biological replicates were analyzed for each tissue and treatment.

### 2.3 Amino acid grouping and amino acid-based nutritional evaluation

Total hydrolyzed amino acid content (TAA) was calculated as the sum of the 18 quantified amino acids. Essential amino acids (EAA) comprised His, Ile, Leu, Lys, Met, Phe, Thr, Trp, and Val, whereas non-essential amino acids (NEAA) were calculated as TAA minus EAA. Branched-chain amino acids (BCAA), sulfur-containing amino acids (SAA), and aromatic amino acids (AAA) were calculated as Val + Ile + Leu, Met + Cys, and Phe + Tyr, respectively. The EAA/TAA and EAA/NEAA ratios were calculated from the corresponding sums.

For protein-normalized evaluation:


$$AA_{i}(\mathrm{mg}\;g^{-1}\;\text{protein})=\frac{\mathrm{AAi}\;(\mathrm{mg}\;g^{-1}\;\mathrm{DW})\times 1000}{\text{Protein}\;(g\;kg^{-1}\;\mathrm{DW})}$$

Amino acid scores (AAS) were calculated as:


$${\mathrm{AAS}}_{i}=\frac{AA_{i}(\mathrm{mg}\;g^{-1}\;\text{protein})}{\mathrm{FAO}\;\text{referenc}e_{i}\;(\mathrm{mg}\;g^{-1}\;\text{protein})}\times 100$$

The FAO reference values for His, Ile, Leu, Lys, Met + Cys, Phe + Tyr, Thr, Trp, and Val were 16, 30, 61, 48, 23, 41, 25, 6.6, and 40 mg g^−1^ protein, respectively (28). The essential amino acid index (EAAI) was calculated as:


$$\text{EAAI}={\left[\prod_{i=1}^{9}(\frac{AA_{i}}{\mathrm{FAO}\;\text{referenc}e_{i}})\right]}^{1/9}\times 100$$

All indices were calculated separately for each biological replicate.

### 2.4 RNA extraction and transcriptomics

Total RNA was extracted using TRIzol reagent and evaluated using an Agilent 5,300 Bioanalyzer and NanoDrop 2000. Libraries prepared using the Illumina Stranded mRNA Prep Kit were sequenced on an Illumina NovaSeq 6000 platform (2 × 150 bp). Reads processed using fastp were aligned to the *M. sextelata* reference genome (ASM2471366v1; GCA_024713665.1) using HISAT2 v2.1.0 and assembled using StringTie. Expression was quantified as transcripts per million (TPM). TPM values were used for PCA, expression visualization, K-means clustering, and WGCNA, whereas raw counts were analyzed using DESeq2. Genes with |log₂ fold change| ≥ 1 and FDR < 0.05 were considered differentially expressed genes (DEGs). KEGG enrichment was performed using KOBAS, with Bonferroni-corrected *p ≤ 0.05* considered significant.

### 2.5 Protein extraction and proteomics

Frozen samples were lysed in 8 M urea containing 1% SDS and protease inhibitors, sonicated, and centrifuged at 12,000 ×*g* for 20 min at 4 °C. Protein concentrations were determined using a ProteoAnalyzer (M5350AA). Protein (100 μg) was reduced with 10 mM tris(2-carboxyethyl)phosphine, alkylated with 40 mM iodoacetamide, and digested overnight with 1:50 (w/w) trypsin. Peptides were desalted, dried, and analyzed using a Vanquish Neo UHPLC coupled to an Orbitrap Astral mass spectrometer (Thermo Fisher Scientific, USA). Separation was performed on a μPAC high-throughput column (75 μm × 5.5 cm) over 8 min, followed by data-independent acquisition in the range of m/z 100–1700.

Raw data were searched against a custom database generated from the transcriptomic dataset using Spectronaut 19. The search settings included trypsin/P, two missed cleavages, fixed cysteine carbamidomethylation, and variable methionine oxidation and protein N-terminal acetylation. Peptide- and protein-level FDRs were controlled at 1%, and protein abundances were quantified using Spectronaut 19. Proteins with >70% missing values were removed, and the remaining missing values were imputed within treatment groups using the k-nearest-neighbor method. Proteins with a fold change ≥1.5 or ≤0.67 and *p* < 0.05 in the two-group t-tests were defined as differentially expressed proteins (DEPs); KEGG enrichment was considered significant at *p* < 0.05.

### 2.6 Expression-pattern clustering and cross-omics analysis

K-means clustering (*k* = 6) was performed separately on transcript TPM and protein-abundance matrices for each tissue. Matched transcript–protein pairs were identified using the correspondence between transcripts and proteins in the custom database and analyzed by Pearson correlation. Positively correlated transcript–protein pairs with *r* > 0.99 and *p* < 0.01 were retained, and the 20 pairs with the highest correlation coefficients were visualized. Cross-omics concordance was assessed by identifying KEGG pathways significantly enriched in both the DEG and DEP datasets within each tissue.

### 2.7 Weighted co-expression network analyses

Weighted gene co-expression network analysis (WGCNA) and weighted protein co-expression network analysis (WPCNA) were performed separately for cap and stipe samples using the WGCNA package (version 1.68) on the Majorbio Cloud platform. TPM expression matrices obtained from the transcriptomic analysis were used for WGCNA, whereas the original protein-abundance matrices were used for WPCNA. Genes with a mean TPM of <1 or a coefficient of variation of <0.1 were filtered out, and 5,777 and 4,490 genes were retained for the cap and stipe networks, respectively. Proteins with a mean abundance of <1 or a coefficient of variation of <0.01 were excluded; 5,635 and 5,582 proteins were retained for the cap and stipe networks, respectively. Signed networks were constructed using soft-thresholding powers of 9 for both transcriptomic networks and 6 and 4 for the cap and stipe proteomic networks, respectively. For all the networks, the minimum module size was 30, the minimum module membershi*p* value for retaining a feature within a module was 0.3, and the module-merging cut height was 0.25. Module eigengenes were correlated with protein and individual hydrolyzed amino acid contents using Spearman correlation, and positively trait-associated modules were subjected to KEGG enrichment analysis. KEGG pathways with *p* < 0.05 for WGCNA modules and adjusted *p* < 0.05 for WPCNA modules were considered significantly enriched. Because each tissue-specific network contained 12 samples, module–trait associations were treated as exploratory statistical associations rather than evidence of causal regulation.

### 2.8 Quantitative real-time PCR (qRT–PCR) validation

Twelve DEGs, comprising six cap DEGs and six stipe DEGs, were selected for qRT–PCR validation. These genes were selected to represent different supplementation-responsive expression patterns and biological processes identified by the transcriptomic and integrated pathway analyses. The feasibility of designing gene-specific primers was also considered. The same RNA samples used for RNA sequencing were used for qRT–PCR validation. cDNA was synthesized from 1 μg of total RNA using PrimeScript RT Master Mix (Perfect Real Time; RR036A, Takara Bio Inc., Japan) on an ABI 2720 thermal cycler (Applied Biosystems, USA), using the program of 37 °C for 15 min followed by 85 °C for 5 s. qRT–PCR was performed using TB Green Premix Ex Taq (Tli RNaseH Plus; RR420A, Takara Bio Inc., Japan) on a 7900HT Sequence Detection System (Applied Biosystems, USA). Each 10-μL reaction contained 5 μL of TB Green Premix, 0.4 μL each of the forward and reverse primers (10 μmol L^−1^), 0.2 μL of ROX Reference Dye II, 1 μL of cDNA, and 3 μL of sterile water. The thermal cycling program consisted of an initial denaturation at 95 °C for 30 s, followed by 40 cycles of 95 °C for 3 s and 60 °C for 30 s. Three biological replicates, each analyzed using three technical replicates, were included. *M. sextelata* actin was used as the reference gene, and the untreated control of the corresponding tissue was used as the calibrator. Relative expression was calculated using the 2^^−ΔΔCt^ method. The selected target genes, reference gene, and primer sequences are listed in Supplementary Table S3.

### 2.9 Statistical analysis

Data are presented as mean ± SD (*n* = 3). Protein and amino acid variables were analyzed across the 12 tissue–treatment groups by one-way ANOVA with Duncan’s test; qRT-PCR data were analyzed separately by tissue. Significance was set at *p* < 0.05 using IBM SPSS Statistics 22.

## 3 Results

### 3.1 Graded soybean meal supplementation differentially alters protein content and amino acid proportionality in *M. sextelata*

Additional soybean meal significantly affected protein content in mature *M. sextelata*, with responses varying by tissue and supplementation level (Figure 1A). In the cap, protein content increased from 303.13 g kg^−1^ DW in CKC to 384.39 g kg^−1^ DW at 8% (26.8%) and declined to 355.03 g kg^−1^ DW at 10%. All supplemented cap groups had higher protein contents than CKC, and the 4 and 8% treatments did not differ significantly. In the stipe, the 1–4% treatments did not differ significantly from CKS; protein content peaked at 8% (354.52 g kg^−1^ DW), 20.2% above CKS, and declined to 328.24 g kg^−1^ DW at 10%. These results demonstrate nonlinear protein responses in both tissues, with the highest contents observed at 8% under the tested conditions.

**Figure 1:**
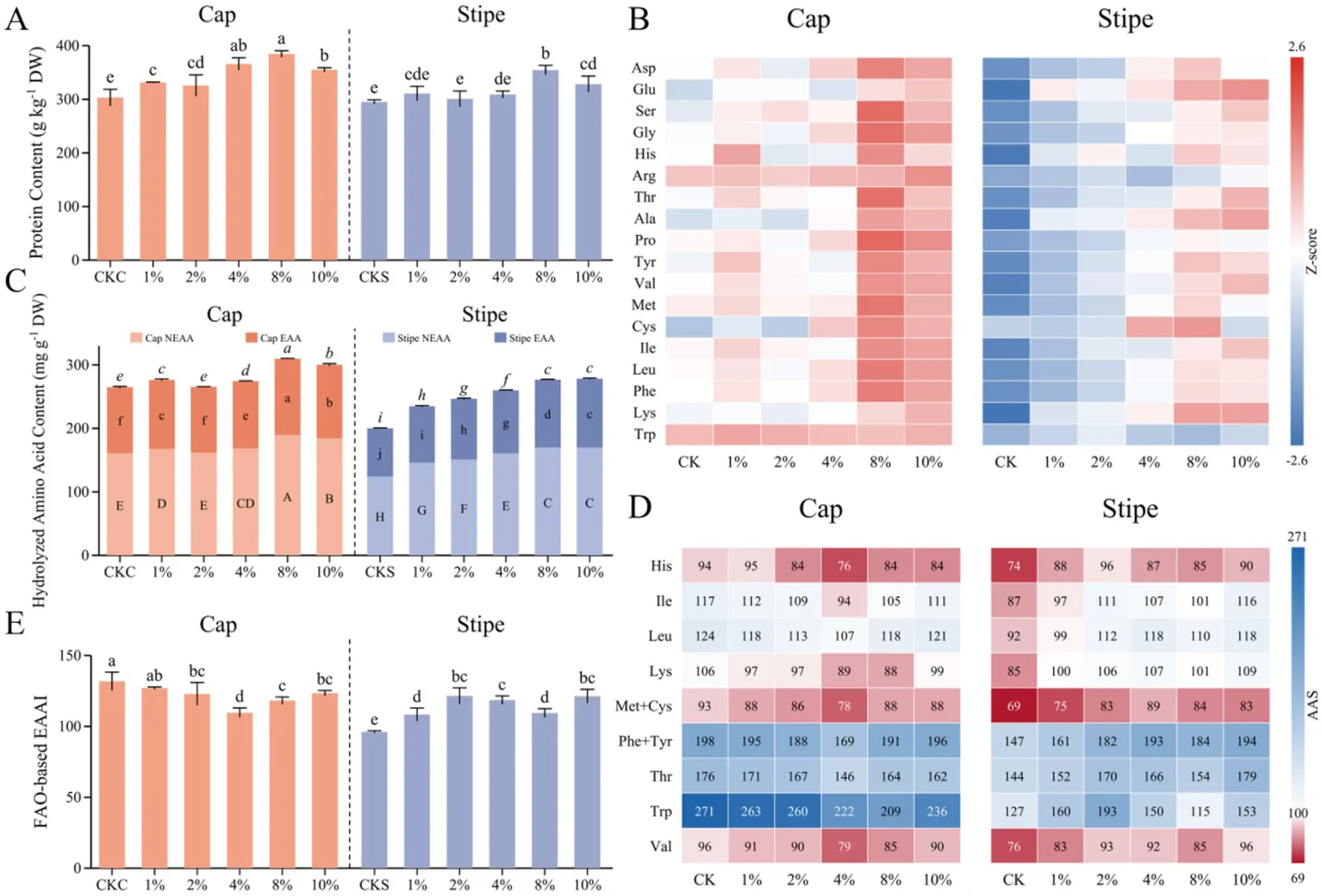
Effects of graded additional soybean meal on protein content, hydrolyzed amino acid composition, and amino acid-based nutritional indices in the cap and stipe of mature *M. sextelata*. The supplementation percentages indicate additional soybean meal relative to the 600-g dry weight of the basal formulation, which already contained 5% soybean meal; they do not represent the total soybean meal proportion in the final formulation. **(A)** Protein contents in the cap and stipe at different supplementation levels. **(B)** Z-score-normalized heatmap of the contents of 18 hydrolyzed amino acids. Blue and red indicate relatively low and high contents, respectively. **(C)** Stacked bar chart of essential amino acid (EAA) and non-essential amino acid (NEAA) contents, with total bar height representing total hydrolyzed amino acids (TAA). **(D)** Heatmap of amino acid scores (AAS) calculated using the FAO reference pattern for individuals aged ≥3 years; a value of 100 indicates equivalence to the corresponding reference value. **(E)** Essential amino acid index (EAAI) calculated using the same FAO reference pattern. CKC and CKS denote the cap and stipe controls without additional soybean meal, respectively. Data in **A**, **C**, and **E** are presented as mean ± SD (*n* = 3). Different letters indicate significant differences among tissue–treatment groups (*p* < 0.05).

The Z-score heatmap showed tissue- and supplementation-level-dependent patterns among the 18 hydrolyzed amino acids (Figure 1B; Supplementary Table S1). Most cap amino acids were relatively high at 8%, whereas Glu, Arg, and Lys peaked at 10%; most stipe amino acids were relatively high at 8% or 10%. TAA, EAA, and NEAA also showed tissue-dependent responses (Figure 1C). In the cap, all three peaked at 8%, reaching 309.81, 120.12, and 189.68 mg g^−1^ DW, respectively—17.0%, 15.6%, and 17.9% above CKC. In the stipe, EAA peaked at 10%, whereas TAA and NEAA did not differ significantly between 8 and 10%. At matched supplementation levels, the cap consistently had higher TAA, EAA, and NEAA contents than the stipe.

AAS values based on the FAO reference pattern for individuals aged ≥3 years differed among the scored amino acid groups (Figure 1D; Supplementary Table S2). Phe + Tyr, Thr, and Trp scores exceeded 100 in all samples, whereas His, Met+Cys, and Val remained below 100 in both tissues. Cap EAAI ranged from 109.64 to 131.80, with the highest value in CKC and the lowest at 4%. Although the 8% treatment had the highest cap protein, TAA, and EAA contents, it did not have the highest EAAI (Figure 1E). In the stipe, all supplemented groups had higher EAAI values than CKS, with the highest numerical values at 2 and 10% (121.54 and 121.31, respectively). Thus, absolute protein and hydrolyzed amino acid contents were not consistently aligned with protein-normalized essential amino acid proportionality, and their relationship differed between tissues.

### 3.2 The cap and stipe exhibit contrasting transcriptomic and proteomic responses to soybean meal supplementation

RNA sequencing of the control, 2%, 4%, and 8% treatments generated 24 libraries and 1,061,847,042 clean reads, with 5.88–7.12 Gb of clean data per library (Supplementary Table S4). Q30 values ranged from 95.50 to 95.90%, and 88.98–95.00% of reads mapped to the reference genome. Transcriptomic PCA separated the tissue–treatment groups, while biological replicates clustered closely; PC1 and PC2 together explained 56.37% of the total variation (Figure 2A). Across the six treatment–control comparisons, 1,406–4,122 DEGs were detected, with upregulated DEGs predominating and more DEGs detected in the cap than in the stipe at each matched supplementation level (Figure 2B). MC caps and LS stipes contained the largest numbers of DEGs within their respective tissues, with 4,122 and 2,120 DEGs, respectively. Only 33 DEGs were shared across all six comparisons, indicating tissue- and supplementation-dependent transcriptional responses (Figure 2C). KEGG enrichment analysis further revealed tissue- and supplementation-level-dependent transcriptional responses (Figure 2D). In the cap, DEGs from LC were enriched mainly in Glycolysis/Gluconeogenesis, Starch and sucrose metabolism, and Tyrosine metabolism, whereas DEGs from MC and HC were enriched mainly in Aminoacyl-tRNA biosynthesis, ribosome-related pathways, and multiple amino acid metabolic pathways. In the stipe, LS DEGs were enriched in amino acid metabolism, Pyruvate metabolism, Purine metabolism, and ribosome-related pathways, whereas MS and HS DEGs were enriched in Aminoacyl-tRNA biosynthesis, Valine, leucine and isoleucine degradation, the Pentose phosphate pathway, and DNA-related processes. These results indicate that the dominant transcriptional responses differed between tissues and shifted with supplementation level.

**Figure 2:**
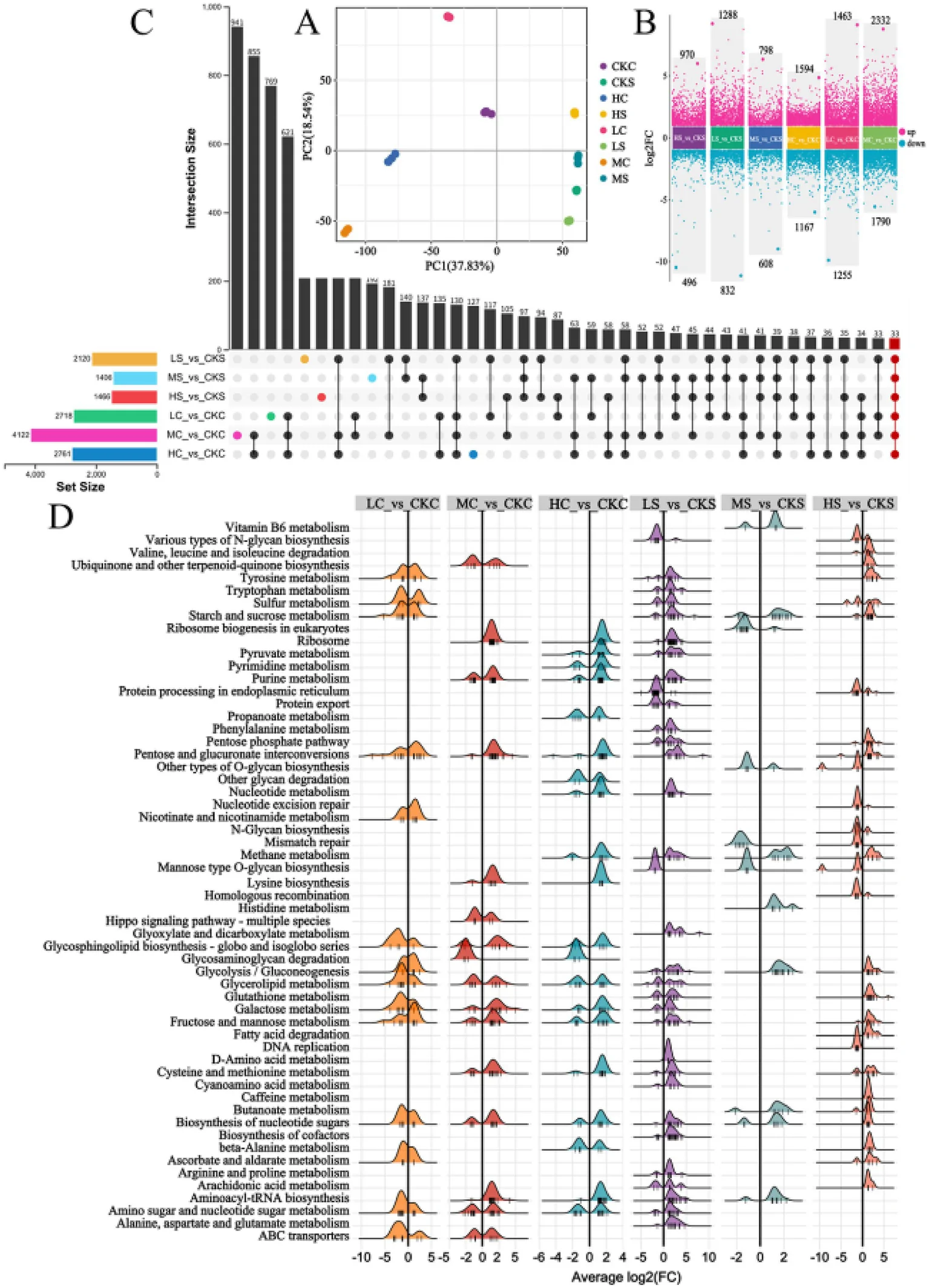
Transcriptomic responses of the cap and stipe of *M. sextelata* to additional soybean meal. **(A)** Principal component analysis (PCA) of transcriptomic profiles across all samples. **(B)** Volcano plots showing differentially expressed genes (DEGs) in each soybean-meal treatment relative to the corresponding tissue control. Pink and blue points indicate significantly upregulated and downregulated genes, respectively. **(C)** UpSet plot showing shared and comparison-specific DEGs among the six treatment–control comparisons. **(D)** Ridge plot of Kyoto Encyclopedia of Genes and Genomes (KEGG) pathway enrichment for DEGs in each comparison. Distributions represent gene-level log₂ fold-change values within each enriched pathway; rightward and leftward shifts indicate predominantly positive and negative expression changes, respectively.

Proteomic analysis quantified 5,969 proteins from 56,179 peptides. Proteomic PCA separated the tissue–treatment groups, while biological replicates clustered closely; PC1 and PC2 explained 41.95 and 11.92% of the total variation, respectively (Figure 3A). At each matched supplementation level, more DEPs were detected in the stipe than in the cap (Figure 3B). Cap comparisons yielded 513–1,001 DEPs, whereas stipe comparisons yielded 752–2,148 DEPs. Downregulated DEPs predominated in both LS and HS, accounting for 1,142 of 1,663 DEPs (68.7%) and 1,358 of 2,148 DEPs (63.2%), respectively. Only 23 DEPs were shared across all six comparisons (Figure 3C). Proteomic KEGG enrichment also varied between tissues and supplementation levels (Figure 3D). In the cap, LC DEPs were enriched mainly in nitrogen metabolism, carbohydrate metabolism, and aromatic amino acid metabolism, whereas HC DEPs showed broader enrichment involving amino acid and glycolytic pathways. In the stipe, LS DEPs were enriched mainly in Oxidative phosphorylation and Spliceosome, MS DEPs in Ribosome biogenesis in eukaryotes, and HS DEPs in Aminoacyl-tRNA biosynthesis, Spliceosome, and Other types of O-glycan biosynthesis. These enrichment patterns indicate that the proteomic response of the stipe involved energy metabolism and RNA-related processes, whereas the cap response was more closely associated with carbon, nitrogen, and amino acid metabolism. Overall, more DEGs were detected in the cap than in the stipe at each matched supplementation level, indicating a broader transcriptional response in the cap. Conversely, more DEPs were detected in the stipe, with downregulated DEPs predominating particularly in the 2 and 8% treatments, indicating a more extensive and predominantly downward proteomic response at these supplementation levels. The two tissues also differed in the biological processes represented by their transcriptomic and proteomic enrichment profiles.

**Figure 3:**
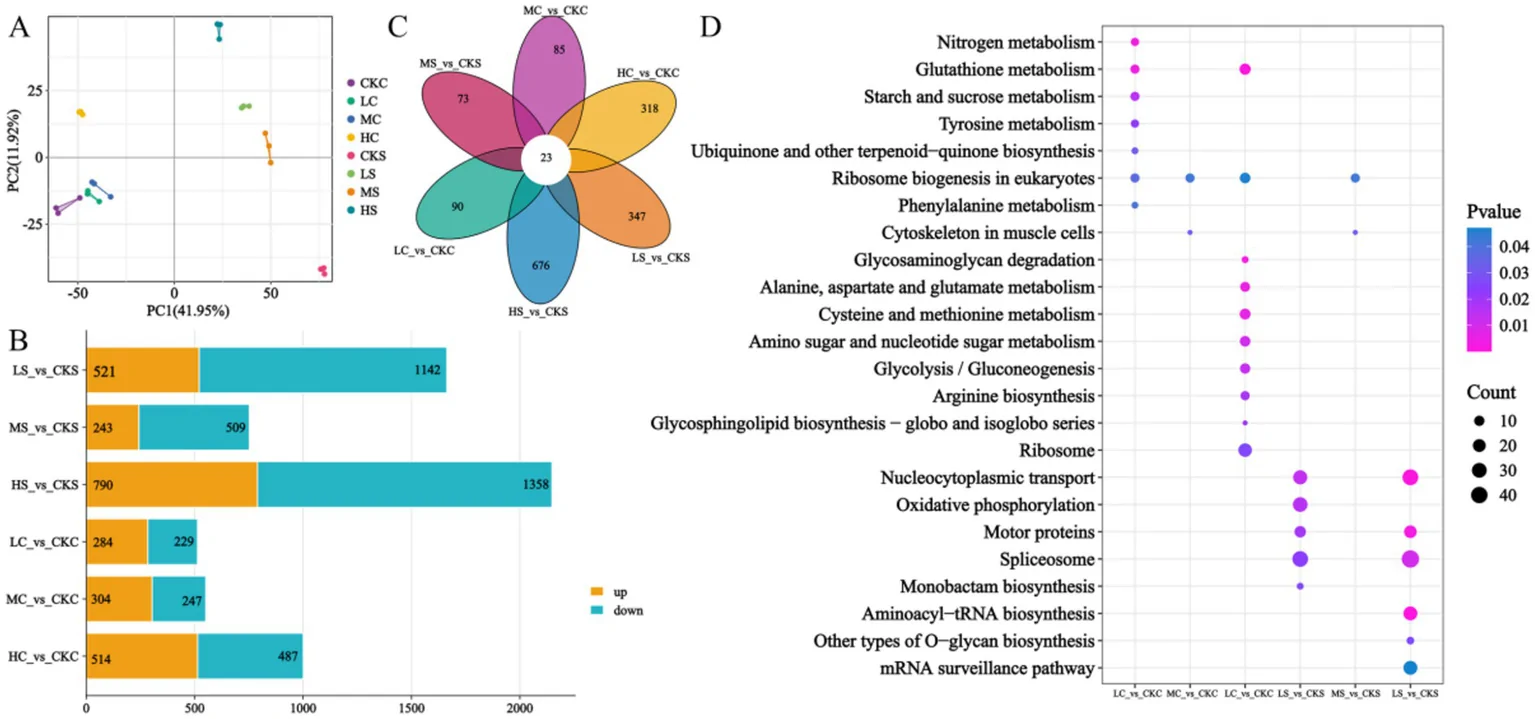
Proteomic responses of the cap and stipe of *M. sextelata* to additional soybean meal. **(A)** Principal component analysis (PCA) of proteomic profiles across all samples. **(B)** Numbers of significantly upregulated (orange) and downregulated (cyan) differentially expressed proteins (DEPs) in each treatment–control comparison. **(C)** Venn diagram showing shared and comparison-specific DEPs among the six comparisons. **(D)** Bubble plot of Kyoto Encyclopedia of Genes and Genomes (KEGG) pathway enrichment for DEPs. Bubble size represents the number of DEPs assigned to each pathway, and color represents the enrichment p value.

### 3.3 Trait-associated gene co-expression modules differ between the cap and stipe

To identify gene co-expression modules associated with soybean-meal-responsive variation in protein and amino acid contents, weighted gene co-expression network analysis (WGCNA) was performed separately on cap and stipe transcriptomes (Figure 4). In the cap, hierarchical clustering revealed seven co-expression modules (Figure 4A). Module–trait analysis revealed distinct associations with nutritional traits (Figure 4B). The 3,528-gene MEturquoise module showed the strongest positive correlations with protein and amino acid contents (coefficients = 0.580–0.965, *p* < 0.05). Conversely, MEred (169 genes) was negatively correlated with all traits (coefficients = −0.615 to −0.944, *p* < 0.05), whereas MEblue, MEbrown, MEgreen, and MEyellow showed no significant correlations. KEGG analysis of MEturquoise genes identified enrichment in Ribosome, Aminoacyl-tRNA biosynthesis, Lysine biosynthesis, Cysteine and methionine metabolism, Valine, leucine and isoleucine biosynthesis, Alanine, aspartate and glutamate metabolism, and Glycine, serine and threonine metabolism (*p* < 0.05; Figure 4C). Cysteine and methionine metabolism therefore recurred across cap DEG, DEP, and trait-associated gene-module analyses. Thus, the principal positively trait-associated cap module was characterized by amino acid metabolism and translation-related pathways.

**Figure 4:**
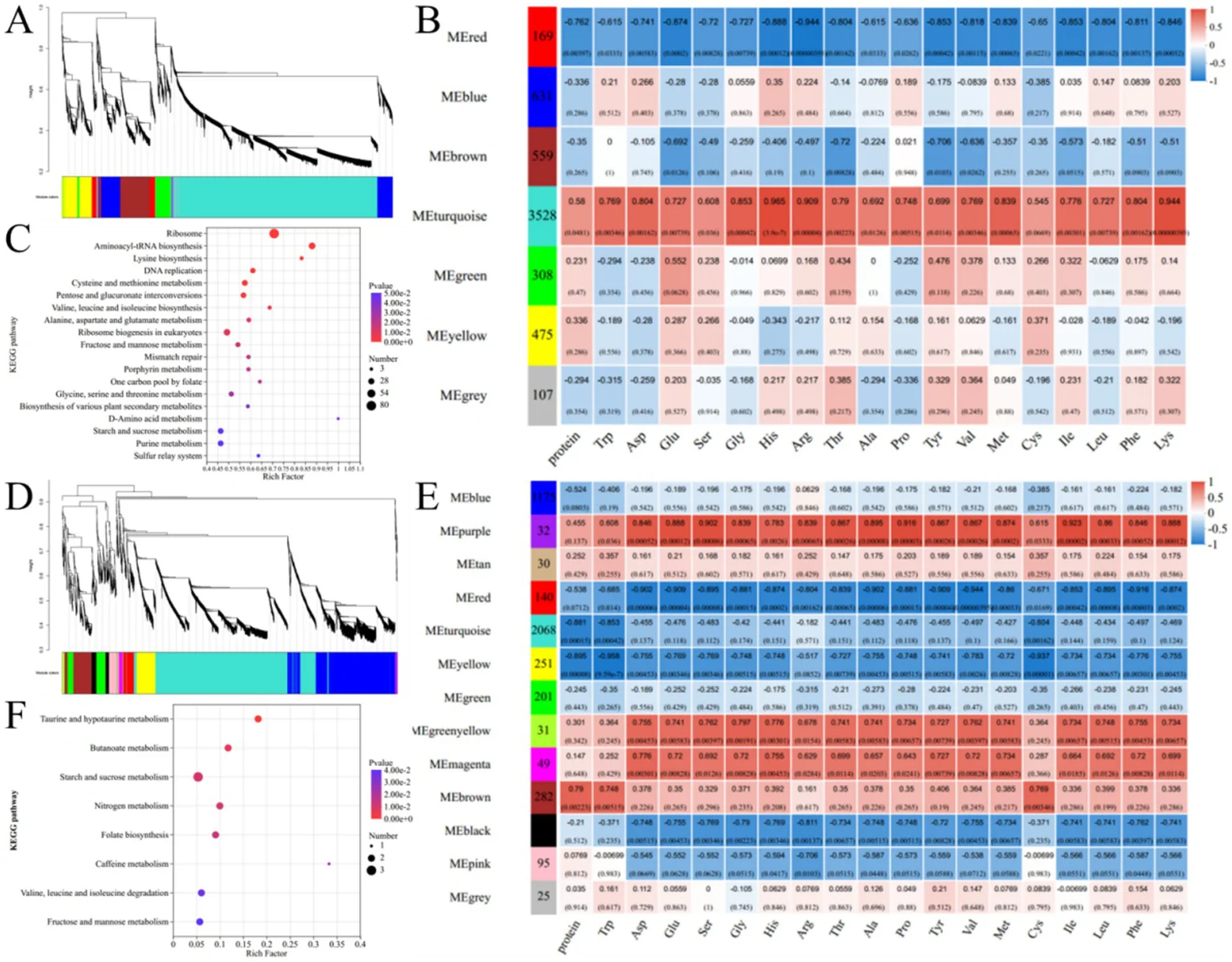
Weighted gene co-expression network analysis (WGCNA) of cap and stipe transcriptomes of *M. sextelata* under additional soybean meal supplementation. **(A,D)** Hierarchical clustering dendrograms of genes in the cap **(A)** and stipe **(D)**, with module assignments indicated by color bars. **(B,E)** Heatmaps of Spearman correlations between module eigengenes and compositional traits in the cap **(B)** and stipe **(E)**. Values indicate correlation coefficients, with corresponding *p* values in parentheses; blue and red represent negative and positive correlations, respectively. **(C,F)** Kyoto Encyclopedia of Genes and Genomes (KEGG) enrichment of genes in the positively trait-associated cap MEturquoise module **(C)** and the combined stipe MEpurple, MEgreenyellow, and MEmagenta modules **(F)**. Bubble size represents the number of genes assigned to each pathway, and color represents the enrichment *p* value.

In the stipe, WGCNA revealed 13 co-expression modules (Figure 4D), among which MEpurple (32 genes), MEgreenyellow (31 genes), and MEmagenta (49 genes) showed significant positive correlations with most individual amino acid contents (coefficients = 0.608–0.916, 0.727–0.797, and 0.629–0.776, respectively; *p* < 0.05; Figure 4E). MEred, MEyellow, and MEblack were negatively correlated with most measured traits (*p* < 0.05). The 112 genes in these three modules were enriched in Starch and sucrose metabolism, Nitrogen metabolism, Valine, leucine and isoleucine degradation, and Fructose and mannose metabolism (*p* < 0.05; Figure 4F). Thus, positively trait-associated stipe modules were characterized mainly by carbohydrate and nitrogen metabolism and branched-chain amino acid degradation.

### 3.4 Trait-associated protein co-expression modules differ between the cap and stipe

Weighted protein co-expression network analysis (WPCNA) was performed separately on cap and stipe proteomes to identify modules associated with nutritional traits under additional soybean meal supplementation (Figure 5). In the cap, hierarchical clustering revealed 21 co-expression modules (Figure 5A), with distinct module–trait associations (Figure 5B). The 537-protein MEyellow module showed significant positive correlations with protein and amino acid contents (coefficients = 0.643–0.863, *p* < 0.05). MEpink and MEpurple were negatively correlated with most nutritional traits, whereas the remaining modules showed no significant correlations. MEyellow proteins were enriched in Aminoacyl-tRNA biosynthesis, Protein processing in endoplasmic reticulum, Pyruvate metabolism, Citrate cycle (TCA cycle), Glycolysis/Gluconeogenesis, Starch and sucrose metabolism, Arginine biosynthesis, and Proteasome (adjusted *p* < 0.05; Figure 5C). Thus, cap protein and amino acid traits were associated with a module characterized by central carbon metabolism, amino acid activation, protein processing, and proteasomal turnover.

**Figure 5:**
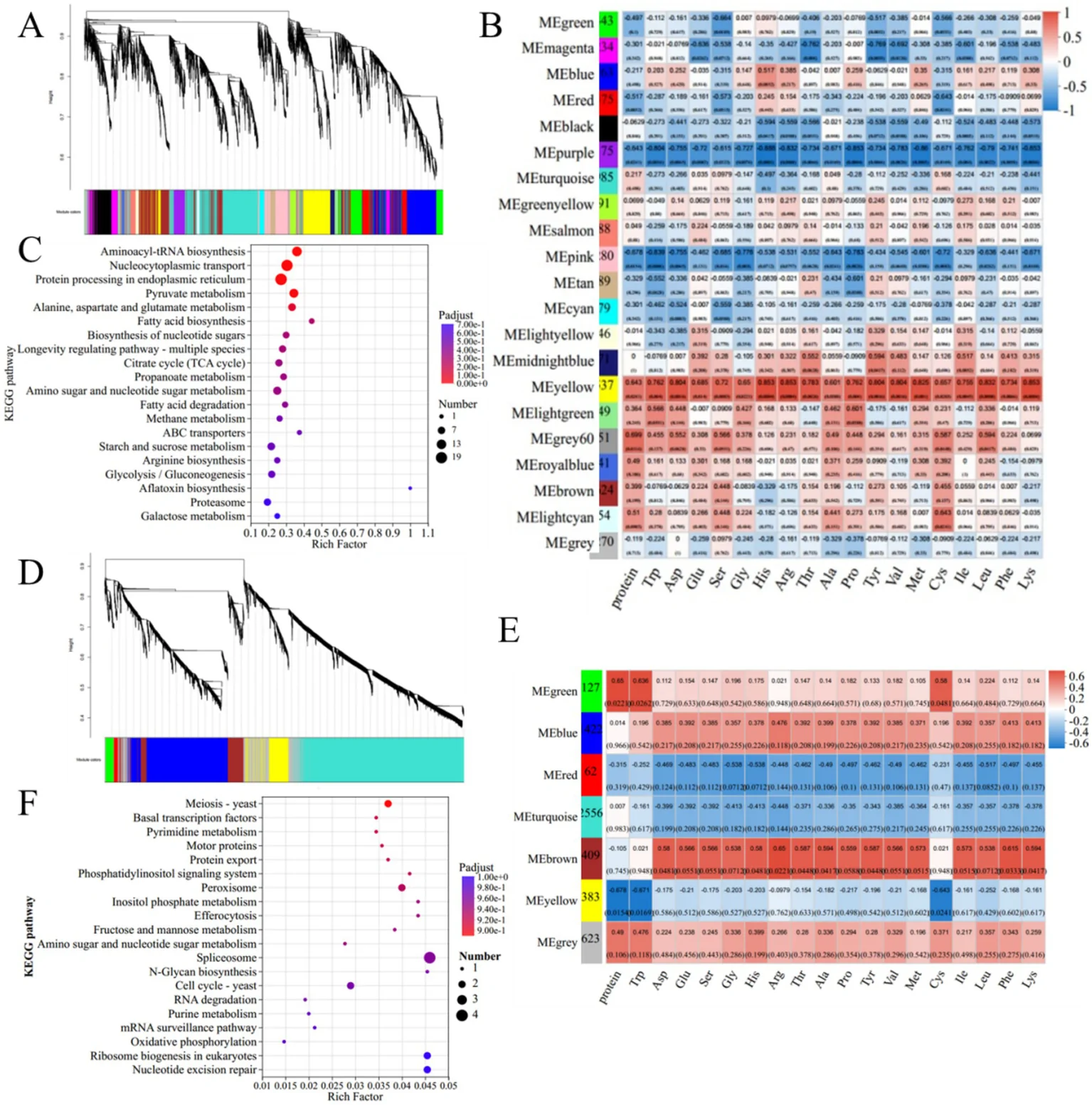
Weighted protein co-expression network analysis (WPCNA) of cap and stipe proteomes of *M. sextelata* under additional soybean meal supplementation. **(A,D)** Hierarchical clustering dendrograms of proteins in the cap **(A)** and stipe **(D)**, with module assignments indicated by color bars. **(B,E)** Heatmaps of Spearman correlations between protein-module eigengenes and compositional traits in the cap **(B)** and stipe **(E)**. Values indicate correlation coefficients, with corresponding *p* values in parentheses; blue and red represent negative and positive correlations, respectively. **(C,F)** Kyoto Encyclopedia of Genes and Genomes (KEGG) enrichment of proteins in the positively trait-associated cap MEyellow module **(C)** and stipe MEbrown module **(F)**. Bubble size represents the number of proteins assigned to each pathway, and color represents the adjusted *p* value.

In the stipe, WPCNA identified seven co-expression modules (Figure 5D) and multiple module–trait associations (Figure 5E). The 409-protein MEbrown module showed significant positive correlations with most amino acid contents (coefficients = 0.580–0.650, *p* < 0.05). MEgreen (127 proteins) was positively correlated with protein, Trp, and Cys contents (coefficients = 0.580–0.650, *p* < 0.05), whereas MEyellow (383 proteins) was negatively correlated with these traits (coefficients = −0.643 to −0.678, *p* < 0.05). MEbrown proteins were enriched in Pyrimidine metabolism, Purine metabolism, Spliceosome, Oxidative phosphorylation, Ribosome biogenesis in eukaryotes, Cell cycle–yeast, mRNA surveillance pathway, and Nucleotide excision repair (adjusted *p* < 0.05; Figure 5F). Unlike cap MEyellow, stipe MEbrown was characterized mainly by oxidative phosphorylation, RNA processing, nucleotide metabolism, and ribosome biogenesis. Together, the gene and protein networks associated cap composition mainly with amino acid and central carbon metabolism, translation, and protein homeostasis, whereas stipe composition was associated with carbohydrate and nitrogen metabolism, amino acid degradation, oxidative phosphorylation, RNA processing, and ribosome biogenesis.

### 3.5 Cross-omics concordance reveals tissue- and concentration-dependent responses

K-means clustering identified transcript and protein clusters with increasing mean abundance across the selected soybean meal levels (Figure 6). In the cap, transcript cluster C5 and protein cluster C5 contained 1,907 genes and 1,031 proteins, respectively; in the stipe, transcript cluster C3 and protein cluster C6 contained 1,337 genes and 918 proteins, respectively (Figures 6A,B,D,E). Within each tissue, positively correlated transcript–protein pairs with *r* > 0.99 and *p* < 0.01 were retained, and the 20 pairs with the highest correlation coefficients were visualized as correlation networks (Figures 6C,F). These networks describe cross-layer expression concordance within the selected clusters but do not establish the regulatory importance of individual nodes.

**Figure 6:**
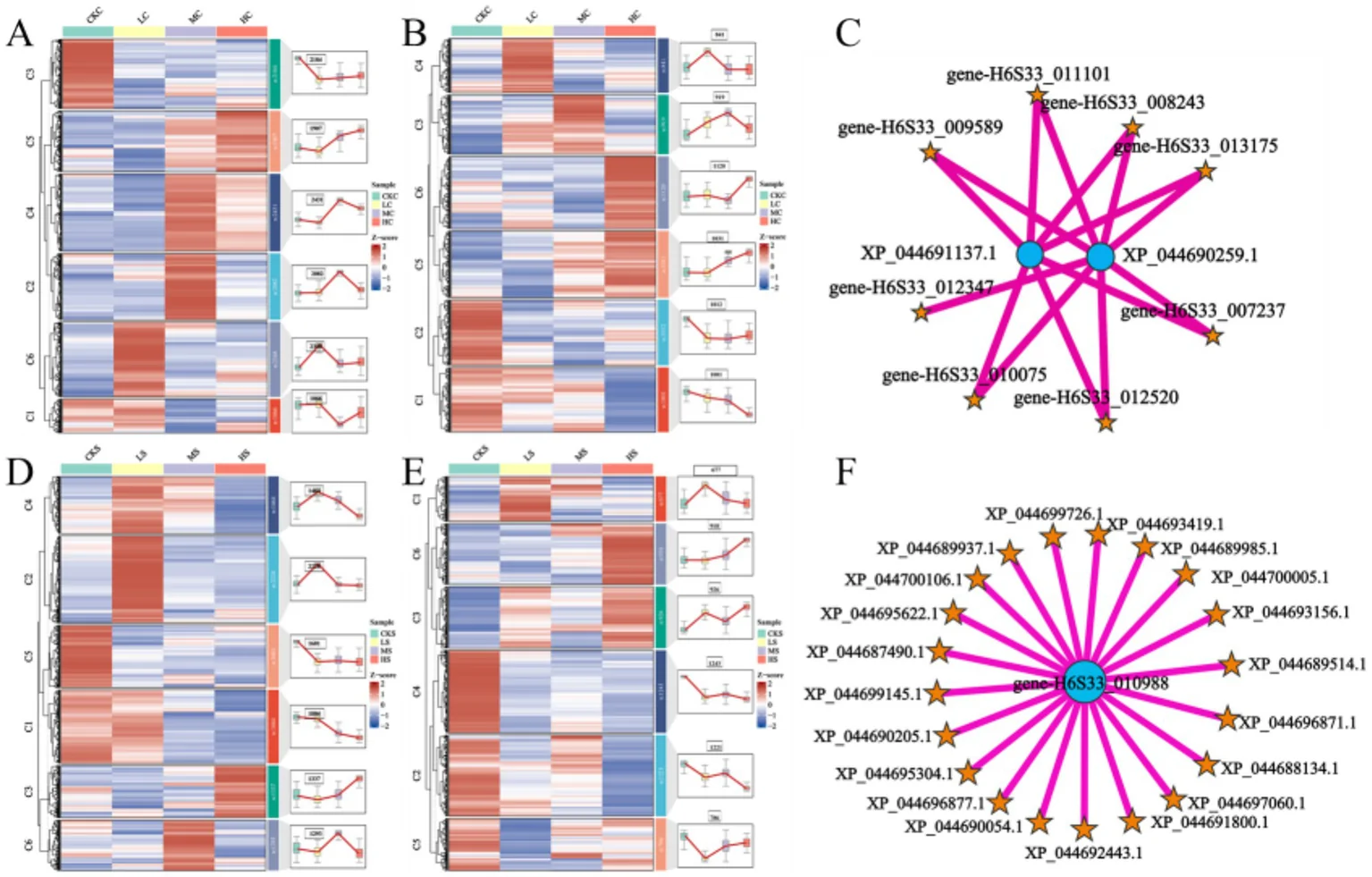
Integrated transcriptomic and proteomic analysis of the cap and stipe of *M. sextelata* under additional soybean meal supplementation. K-means clustering heatmaps of Z-score-normalized transcript-expression and protein-abundance profiles in the cap **(A,B)** and stipe **(D,E)**, respectively, partitioned into six clusters (C1–C6). Line plots on the right show the mean patterns of each cluster across treatment groups. Correlation networks show the 20 matched transcript–protein pairs with the highest positive Pearson correlation coefficients in the cap **(C)** and stipe **(F)**, selected using the thresholds of *r* > 0.99 and *p* < 0.01.

Cross-omics KEGG analysis identified nine pathways independently enriched in both the cap transcriptomic and proteomic datasets: Glycolysis/Gluconeogenesis, Amino sugar and nucleotide sugar metabolism, Glycosphingolipid biosynthesis–globo and isoglobo series, Starch and sucrose metabolism, Tyrosine metabolism, Ribosome, Ubiquinone and other terpenoid-quinone biosynthesis, Glycosaminoglycan degradation, and Cysteine and methionine metabolism (Figure 7A). Among the amino-acid-related pathways, Cysteine and methionine metabolism also recurred in the positively trait-associated cap transcript module, making it the most consistently represented amino-acid-related pathway across the cap analyses; Tyrosine metabolism represented an additional transcriptome–proteome overlap. In the stipe, Ribosome biogenesis in eukaryotes, Aminoacyl-tRNA biosynthesis, and Other types of O-glycan biosynthesis were enriched in both datasets (Figure 7B). Thus, cross-omics pathway concordance involved amino acid, carbohydrate, and ribosome-related processes in the cap, but mainly ribosome biogenesis, amino acid activation, and glycan biosynthesis in the stipe.

**Figure 7:**
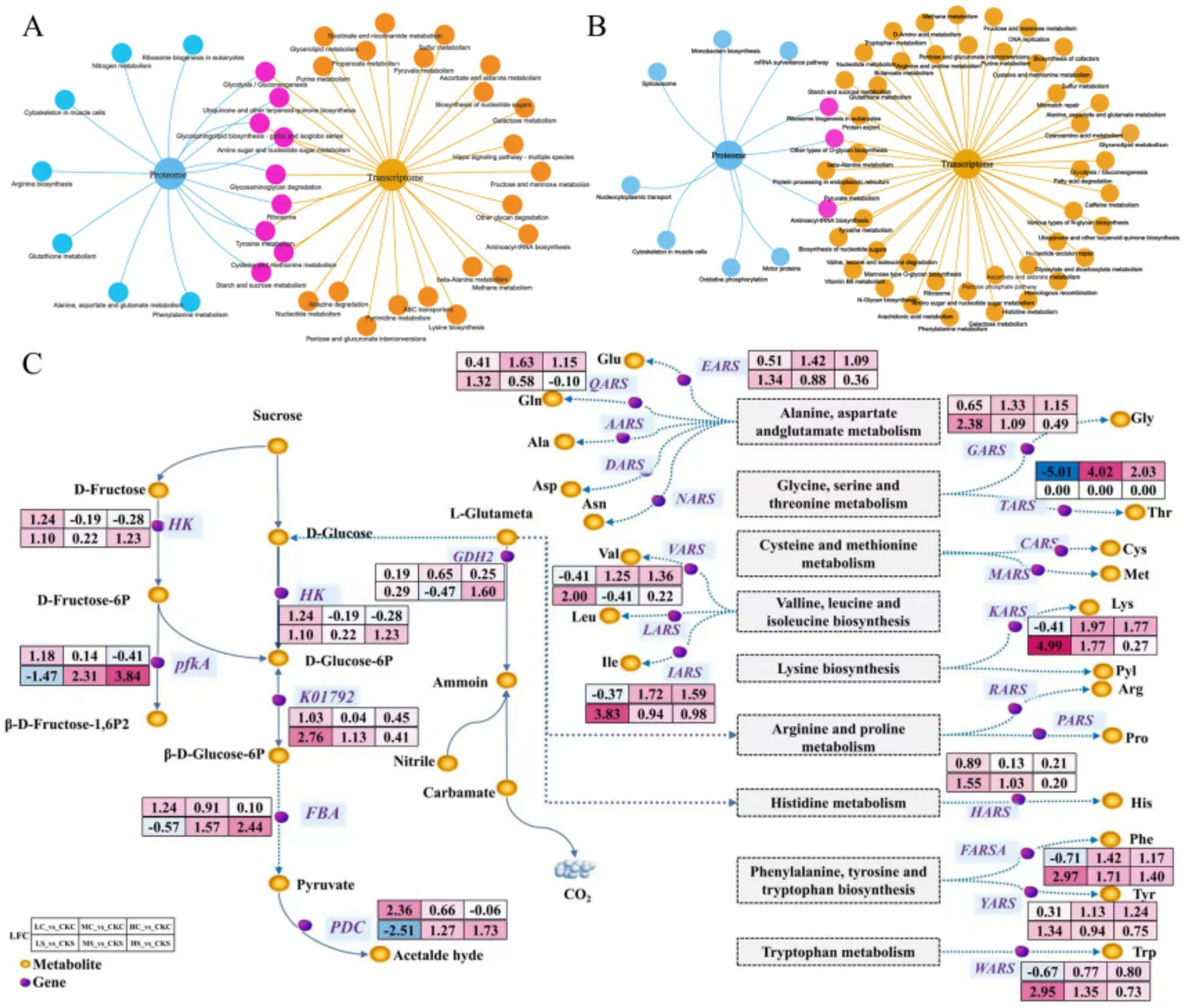
Cross-omics pathway concordance in the cap and stipe of *M. sextelata* under additional soybean meal supplementation. Kyoto Encyclopedia of Genes and Genomes (KEGG) pathways independently enriched in both the transcriptomic and proteomic datasets of the cap **(A)** and stipe **(B)**. Orange and blue nodes represent pathways identified in the transcriptomic and proteomic datasets, respectively, whereas pink nodes represent pathways shared between the two omics layers. **(C)** Integrated pathway map connecting measured compositional traits with treatment-responsive genes involved in central carbon, nitrogen, and amino acid-related processes. Values adjacent to selected genes indicate log₂ fold changes relative to the corresponding tissue control across the selected soybean meal levels.

Expression patterns in the integrated gene–pathway network differed among supplementation levels (Figure 7C). Glycolysis-related genes, including *HK*, *pfkA*, *FBA*, K01792, and *PDC*, generally showed their strongest responses in LC caps but in MS or HS stipes, while GDH2 responded most strongly in HS stipes. In the cap, several aminoacyl-tRNA synthetase genes, including *QARS*, *VARS*, *IARS*, and *KARS*, generally responded more strongly in MC and HC. In particular, the log₂FC values of *VARS* and *YARS* progressively increased from LC to HC, while hydrolyzed Val and Tyr contents were significantly higher in 8% caps than in CKC (Figures 1B, 7C; Supplementary Table S1). These parallel trends linked Val and Tyr composition with the expression of the corresponding aminoacyl-tRNA synthetase genes. By contrast, most amino-acid-related genes in the stipe responded most strongly in LS. Overall, the cap showed stronger glycolysis-related responses at the low level and stronger aminoacyl-tRNA-synthetase-related responses at the medium and high levels, whereas the stipe showed stronger amino-acid-related responses at the low level and stronger glycolysis-related responses at the medium and high levels. qRT-PCR analysis of 12 selected DEGs generally supported the direction and broad concentration-response trends observed by RNA sequencing, providing qualitative validation of the transcriptomic results (Supplementary Figure S1).

## 4 Discussion

### 4.1 Soybean meal supplementation produces nonlinear protein responses and distinct essential amino acid proportionality

Protein content reached its highest measured value at 8% supplementation in both the cap and stipe but was lower at 10% (Figure 1A; Supplementary Table S1). In the cap, however, protein content did not differ significantly between the 4 and 8% treatments (Figure 1A). Thus, 8% supplementation produced the highest observed protein content under the tested conditions, but the response did not increase proportionally with soybean meal supplementation and should not be interpreted as evidence of a universally optimal formulation. Edible mushroom-forming fungi convert substrate constituents into fungal biomass with species- and substrate-dependent efficiencies (18, 29). Substrate-dependent differences in protein and metabolite profiles have also been reported in *Pleurotus ostreatus* (30), whereas greater nitrogen input in button-mushroom compost did not necessarily increase nitrogen recovery in fruiting bodies (31), and intensive nutrient amendment in oyster mushroom cultivation did not consistently increase fruiting-body protein content (32). Together with the present results, these findings indicate that increasing a protein-rich substrate component does not necessarily produce a proportional increase in harvested mushroom protein. One plausible explanation is that soybean-meal constituents must first be released from the substrate and assimilated by the mycelium before contributing to fruiting-body composition, and these processes are influenced by fungal extracellular degradation and nutrient-utilization regulation (33, 34). This nonlinear response indicates that supplementation intensity and compositional benefit cannot be assumed to increase proportionally, supporting the evaluation of graded rather than single supplementation levels.

At the higher supplementation levels, increases in protein, TAA, and EAA contents were not consistently accompanied by increases in EAAI. This divergence reflects the different calculation bases of these measurements. Protein and EAA contents represent amounts per unit fruiting-body dry matter, whereas EAAI is calculated from individual essential amino acid contents normalized to protein content. Protein content and amino acid-based nutritional indices therefore describe related but distinct dimensions of mushroom protein composition (35). EAAI can therefore remain unchanged or decline when protein content increases proportionally more than the essential amino acids or when individual essential amino acids respond unevenly. In the cap, the 8% treatment produced the highest protein, TAA, and EAA contents per unit dry weight (Figures 1A,C; Supplementary Table S1), whereas its EAAI remained below that of CKC (Figure 1E; Supplementary Table S2). In contrast, soybean meal supplementation produced a different EAAI response in the stipe, indicating that the proportional distribution of essential amino acids relative to protein was tissue dependent (Figure 1E; Supplementary Table S2). This tissue dependence is consistent with previously reported compositional differences between the cap and stipe of *M. sextelata* (10). Therefore, increased protein and essential amino acid abundance per unit dry matter does not necessarily indicate improved protein-normalized essential amino acid proportionality, and these measurements should be considered complementary rather than interchangeable indicators of composition.

### 4.2 Compositional changes are associated with carbon–nitrogen metabolism, translation, and protein homeostasis

The molecular processes associated with the compositional responses differed between tissues and omics layers. In the cap, transcriptomic enrichment shifted from carbohydrate-related pathways at the low supplementation level to amino acid metabolism, aminoacyl-tRNA biosynthesis, and ribosome-related pathways mainly at the medium and high levels (Figure 2D). The positively trait-associated MEturquoise gene module was enriched in amino acid metabolism, aminoacyl-tRNA biosynthesis, and the ribosome (Figures 4B,C), whereas the positively trait-associated MEyellow protein module included glycolysis, the TCA cycle, pyruvate metabolism, aminoacyl-tRNA biosynthesis, endoplasmic reticulum protein processing, and the proteasome (Figures 5B,C). These patterns are biologically connected because central carbon metabolism supplies energy and carbon skeletons for amino acid metabolism, while glutamate and glutamine participate in fungal nitrogen assimilation and amino-group transfer (34, 36). Coordination between nitrogen status and mitochondrial pyruvate transport or the TCA cycle has been reported in *Ganoderma lucidum* (37), and associations between carbohydrate utilization and amino acid metabolism have been observed following substrate reformulation in other cultivated fungi (25, 38). Thus, the present enrichment and co-expression results associate cap composition with carbon-skeleton supply, amino acid utilization, translation, and protein turnover, although they do not establish causal regulation by these processes.

The incorporation of amino acids into proteins requires amino acid activation, tRNA charging, ribosomal translation, folding, and protein quality control. Aminoacyl-tRNA synthetases connect amino acid availability with translation by attaching amino acids to their cognate tRNAs (22). In the present study, aminoacyl-tRNA biosynthesis and ribosome-related processes were enriched in the cap gene module associated with protein and amino acid traits (Figures 4B,C), suggesting that the compositional response was accompanied by adjustments in translation-related processes. At the protein level, endoplasmic reticulum protein processing and the proteasome were enriched in the corresponding trait-associated module (Figures 5B,C), extending this association to protein folding, maturation, turnover, and recycling. Stage-dependent regulation of ribosome production, protein synthesis, and protein degradation has also been observed during fruiting-body development in Agaricomycetes (39), while fungal endoplasmic-reticulum-associated degradation and related quality-control systems contribute to proteome homeostasis (20, 40, 41). Therefore, the combined transcriptomic and proteomic associations place the cap compositional response in the context of several stages of protein homeostasis rather than translation alone.

Val and Tyr provided a more specific correspondence between hydrolyzed amino acid composition and transcriptomic responses. Both amino acids reached their highest contents in the 8% cap treatment (Figure 1B; Supplementary Table S1), while the log₂FC values of VARS and YARS increased from LC to HC in the integrated network (Figure 7C). These parallel treatment-dependent trends are consistent with, but do not prove, coordinated changes in amino acid composition and aminoacylation-related gene expression. Val biosynthesis uses pyruvate-derived carbon skeletons, whereas Tyr biosynthesis depends on aromatic amino acid precursors derived from central carbon metabolism. The coordination of carbon and nitrogen utilization and the reported effects of soybean-derived nutrients on fungal glycolysis, pyruvate metabolism, and amino acid metabolism provide a plausible metabolic context for these responses (34, 42). Cysteine and methionine metabolism was shared between the cap transcriptomic and proteomic enrichment results (Figure 7A) and was also enriched in the positively trait-associated MEturquoise gene module (Figure 4C). Together with the highest Cys and Met contents in the 8% cap treatment (Figure 1B; Supplementary Table S1), these results associate the cap compositional response with sulfur amino acid metabolism. Because Cys and Met participate in protein synthesis, methyl-group metabolism, glutathione production, and redox homeostasis (43, 44), this pathway may represent a biologically relevant component of the cap response. Beyond these intracellular functions, sulfur amino acid metabolism may also have implications for mushroom aroma through its potential relationship with the formation of volatile sulfur compounds. In *Lentinula edodes*, sulfur-containing volatiles, including lenthionine, 1,2,4-trithiolane, dimethyl disulfide, and dimethyl trisulfide, are important components of the volatile profile and vary markedly among fruiting-body tissues and strains (45). In *Boletus edulis* during processing, changes in sulfur-containing volatile compounds were associated with γ-glutamyl transpeptidase and C–S lyase activities and with transformations of amino acid-related metabolites (46), providing a possible mechanistic precedent for a connection between sulfur metabolism and mushroom aroma. However, these findings were obtained from other mushroom species and, in the case of *B. edulis*, under processing conditions. Moreover, the present study quantified hydrolyzed amino acids rather than free sulfur amino acids, nonprotein sulfur precursors, enzyme activities, volatile compounds, or sensory attributes. Targeted measurements of free sulfur amino acids, relevant enzyme activities, sulfur-containing volatiles, and sensory properties will be required to evaluate this possibility in *M. sextelata*.

### 4.3 The cap and stipe exhibit distinct molecular response architectures

Across matched treatments, the cap generally contained higher protein, TAA, EAA, and NEAA contents than the stipe (Figures 1A,C; Supplementary Table S1), and more DEGs were detected in the cap at each matched supplementation level (Figure 2B). The positively trait-associated cap gene module was characterized mainly by amino acid metabolism, aminoacyl-tRNA biosynthesis, and ribosome-related pathways (Figures 4B,C), whereas the positively trait-associated cap protein module additionally involved glycolysis, the TCA cycle, endoplasmic reticulum protein processing, and the proteasome (Figures 5B,C). These complementary associations indicate that cap composition was associated with molecular processes related to amino acid and central carbon metabolism, translation, and protein homeostasis. The broader transcriptional response of the cap is consistent with previously reported compositional differences between the cap and stipe of *M. sextelata* (10) and with extensive transcriptional regulation during the differentiation and maturation of spore-bearing tissues in mushroom-forming fungi (39, 47). Thus, the cap response combined greater compositional abundance with broader transcriptional changes, although the present analyses do not establish that these molecular processes caused the observed compositional differences.

The stipe exhibited a contrasting molecular profile. At each matched supplementation level, more DEPs were detected in the stipe than in the cap (Figure 3B). Downregulated DEPs predominated in both LS and HS, accounting for 68.7 and 63.2% of the DEPs, respectively (Figure 3B). The occurrence of this pattern at both low and high supplementation levels suggests that the predominantly downward proteomic response did not show a simple correspondence with the highest protein content observed at 8% supplementation. Proteomic enrichment and trait-association analyses further linked the stipe response to carbohydrate and nitrogen metabolism, branched-chain amino acid degradation, oxidative phosphorylation, RNA processing, and ribosome biogenesis (Figures 3D, 4E,F, 5E,F). Tissue-resolved multi-omics analysis of *Pleurotus giganteus* similarly identified prominent nitrogen and amino acid metabolism in the stipe and differences in carbon utilization between the cap and stipe (26). Moreover, RNA processing and ribosome biogenesis also form part of the molecular reorganization associated with tissue differentiation and fruiting-body development in Agaricomycetes (39), while tissue-resolved analysis of *Dictyophora indusiata* revealed distinct molecular and metabolic profiles among differentiated fruiting-body tissues (27). Although these previous studies support substantial molecular differentiation among mushroom fruiting-body tissues, whether nutrient supplementation generally induces a predominantly downward proteomic response in the stipe remains unclear.

## 5 Conclusion

Graded soybean meal supplementation produced nonlinear and tissue-dependent changes in the protein and hydrolyzed amino acid composition of mature *M. sextelata*. Under the tested conditions, 8% supplementation produced the highest measured protein content in both tissues and the highest TAA and EAA contents in the cap, but it did not maximize cap EAAI. Thus, increased compositional abundance and improved protein-normalized essential amino acid proportionality should not be treated as equivalent nutritional outcomes. The cap displayed broader transcriptional responses associated with amino acid and central carbon metabolism, translation, and protein homeostasis, whereas the stipe displayed more extensive proteomic changes associated with carbon and nitrogen metabolism, energy metabolism, amino acid turnover, and RNA processing. These findings provide a tissue-resolved basis for evaluating preharvest nutrient formulations in cultivated morels. Future studies integrating free amino acid profiling, enzyme-activity assays, metabolic-flux analysis, and volatile and sensory measurements are needed to determine whether the identified molecular associations translate into functional nutritional or flavor-related changes.

## Data Availability

The raw RNA-sequencing data have been deposited in the NCBI Sequence Read Archive (SRA) under BioProject accession PRJNA1454502. The raw proteomic data have been deposited in OMIX, National Genomics Data Center, China National Center for Bioinformation, under accession OMIX019234.
