# Supplementary material for: Preharvest soybean meal supplementation alters tissue-specific protein and amino acid composition in Morchella sextelata: integrated transcriptomic and proteomic analysis
Source: Front Nutr. 2026 Oct 19;13:1979592. doi: 10.3389/fnut.2026.1979592 (PMC13724355; doi:10.3389/fnut.2026.1979592)
Supplement: Supplementary file 1 [file Supplementary_file_1.DOCX]

Supplementary Information

Table S1 Protein content, hydrolyzed amino acid composition, and derived nutritional indices of the cap and stipe of Morchella sextelata under different levels of additional soybean meal.

| Variable | Cap | | | | | | Stipe | | | | | |
| --- | --- | --- | --- | --- | --- | --- | --- | --- | --- | --- | --- | --- |
|  | CKC | 1% | 2% | 4% | 8% | 10% | CKS | 1% | 2% | 4% | 8% | 10% |
| Protein (g/kg DW) | 303.13 ± 15.57 e | 331.30 ± 0.86 c | 325.53 ± 19.95cd | 365.99 ± 11.41ab | 384.39 ± 6.05 a | 355.03 ± 3.80 b | 295.04 ± 3.89 e | 310.67 ± 13.28 cde | 300.69 ± 14.79 e | 309.06 ± 6.32 de | 354.52 ± 8.59 b | 328.24 ± 15.07cd |
| Asp (mg/g DW) | 31.09 ± 0.45 e | 32.09 ± 0.76 d | 30.17 ± 0.29 f | 32.85 ± 0.40 c | 35.57 ± 0.42 a | 34.33 ± 0.45 b | 25.24 ± 0.37 i | 27.82 ± 0.46 h | 28.54 ± 0.11 g | 31.69 ± 0.50 de | 33.22 ± 0.30 c | 31.23 ± 0.26 e |
| Glu (mg/g DW) | 32.69 ± 0.27 h | 34.26 ± 0.81 ef | 34.29 ± 0.24 ef | 33.23 ± 0.09 gh | 35.44 ± 0.48 cd | 36.18 ± 0.65 bc | 28.33 ± 0.40 i | 35.00 ± 0.99 de | 33.96 ± 0.30 fg | 35.26 ± 0.51 cd | 36.96 ± 0.38 ab | 37.67 ± 0.53 a |
| Ser (mg/g DW) | 14.04 ± 0.34 e | 14.92 ± 0.18 cd | 15.20 ± 0.24 c | 14.81 ± 0.24 d | 17.24 ± 0.26 a | 15.96 ± 0.17 b | 11.64 ± 0.07 g | 13.02 ± 0.09 f | 14.03 ± 0.16 e | 14.07 ± 0.22 e | 14.92 ± 0.12 cd | 15.61 ± 0.23 b |
| Gly (mg/g DW) | 17.93 ± 0.27 f | 18.66 ± 0.47 de | 17.62 ± 0.39 f | 19.52 ± 0.39 c | 23.17 ± 0.57 a | 21.68 ± 0.32 b | 12.57 ± 0.13 i | 15.00 ± 0.25 h | 15.75 ± 0.31 g | 18.13 ± 0.10 ef | 18.75 ± 0.30 d | 18.96 ± 0.27 cd |
| His (mg/g DW) | 4.54 ± 0.04 fg | 5.05 ± 0.06 b | 4.38 ± 0.05 hi | 4.44 ± 0.06 gh | 5.18 ± 0.01 a | 4.77 ± 0.07 cd | 3.48 ± 0.05 j | 4.35 ± 0.08 hi | 4.63 ± 0.05 ef | 4.32 ± 0.07 i | 4.84 ± 0.07 c | 4.71 ± 0.07 de |
| Arg (mg/g DW) | 14.82 ± 0.19 de | 14.97 ± 0.14 cd | 14.61 ± 0.25 e | 15.19 ± 0.14 bc | 15.35 ± 0.18 b | 16.38 ± 0.09 a | 9.32 ± 0.12 j | 10.60 ± 0.12 h | 11.63 ± 0.14 g | 10.27 ± 0.02 i | 11.68 ± 0.20 g | 12.79 ± 0.15 f |
| Thr (mg/g DW) | 13.29 ± 0.23 e | 14.13 ± 0.20 c | 13.54 ± 0.11 de | 13.35 ± 0.16 e | 15.75 ± 0.27 a | 14.42 ± 0.20 bc | 10.63 ± 0.09 h | 11.83 ± 0.19 g | 12.73 ± 0.22 f | 12.82 ± 0.13 f | 13.68 ± 0.18 d | 14.66 ± 0.12 b |
| Ala (mg/g DW) | 19.92 ± 0.22 e | 20.43 ± 0.48 de | 19.90 ± 0.25 e | 21.14 ± 0.25 c | 23.28 ± 0.23 a | 22.74 ± 0.36 ab | 16.59 ± 0.20 f | 20.43 ± 0.32 de | 20.57 ± 0.48 d | 21.47 ± 0.39 c | 22.63 ± 0.09 b | 23.16 ± 0.34 ab |
| Pro (mg/g DW) | 19.69 ± 0.17 e | 20.40 ± 0.28 d | 18.94 ± 0.24 f | 21.25 ± 0.19 c | 26.55 ± 0.29 a | 24.72 ± 0.31 b | 12.35 ± 0.14 i | 14.86 ± 0.18 h | 16.21 ± 0.15 g | 18.78 ± 0.21 f | 19.95 ± 0.14 e | 18.83 ± 0.21 f |
| Tyr (mg/g DW) | 10.63 ± 0.07 f | 11.85 ± 0.17 c | 10.99 ± 0.08 e | 10.60 ± 0.02 f | 12.85 ± 0.12 a | 12.21 ± 0.12 b | 8.01 ± 0.06 i | 9.24 ± 0.10 h | 10.11 ± 0.13 g | 10.78 ± 0.11 f | 11.88 ± 0.14 c | 11.48 ± 0.22 d |
| Val (mg/g DW) | 11.59 ± 0.19 ef | 12.06 ± 0.09 d | 11.73 ± 0.15 e | 11.51 ± 0.17 ef | 13.13 ± 0.21 a | 12.85 ± 0.10 b | 8.95 ± 0.07 i | 10.34 ± 0.06 h | 11.13 ± 0.05 g | 11.34 ± 0.25 fg | 12.10 ± 0.19 d | 12.58 ± 0.09 c |
| Met (mg/g DW) | 6.38 ± 0.05 d | 6.60 ± 0.15 c | 6.31 ± 0.07 d | 6.40 ± 0.08 cd | 7.54 ± 0.10 a | 6.99 ± 0.09 b | 4.55 ± 0.05 h | 5.27 ± 0.03 g | 5.63 ± 0.06 f | 6.14 ± 0.08 e | 6.62 ± 0.14 bc | 6.13 ± 0.07 e |
| Cys (mg/g DW) | 0.11 ± 0.00 h | 0.14 ± 0.00 e | 0.12 ± 0.00 g | 0.19 ± 0.00 d | 0.23 ± 0.01 a | 0.20 ± 0.00 c | 0.12 ± 0.00 g | 0.12 ± 0.00 g | 0.13 ± 0.00 f | 0.21 ± 0.00 c | 0.22 ± 0.00 b | 0.13 ± 0.00 f |
| Ile (mg/g DW) | 10.59 ± 0.10 e | 11.12 ± 0.15 d | 10.63 ± 0.15 e | 10.33 ± 0.11 f | 12.14 ± 0.07 a | 11.84 ± 0.09 b | 7.72 ± 0.16 i | 9.04 ± 0.19 h | 9.96 ± 0.05 g | 9.92 ± 0.09 g | 10.77 ± 0.07 e | 11.37 ± 0.13 c |
| Leu (mg/g DW) | 22.83 ± 0.66 d | 23.75 ± 0.33 c | 22.32 ± 0.33 d | 23.92 ± 0.35 c | 27.61 ± 0.42 a | 26.22 ± 0.41 b | 16.49 ± 0.25 g | 18.70 ± 0.10 f | 20.44 ± 0.34 e | 22.32 ± 0.47 d | 23.82 ± 0.34 c | 23.61 ± 0.28 c |
| Phe (mg/g DW) | 13.91 ± 0.12 d | 14.64 ± 0.28 c | 13.98 ± 0.14 d | 14.75 ± 0.21 c | 17.20 ± 0.14 a | 16.25 ± 0.05 b | 9.72 ± 0.08 g | 11.20 ± 0.17 f | 12.29 ± 0.18 e | 13.67 ± 0.16 d | 14.81 ± 0.30 c | 14.57 ± 0.24 c |
| Lys (mg/g DW) | 15.33 ± 0.23 d | 15.49 ± 0.38 cd | 15.15 ± 0.10 de | 15.54 ± 0.18 cd | 16.26 ± 0.22 b | 16.88 ± 0.27 a | 12.03 ± 0.10 f | 14.84 ± 0.13 e | 15.21 ± 0.12 de | 15.84 ± 0.13 c | 17.21 ± 0.27 a | 17.21 ± 0.32 a |
| Trp (mg/g DW) | 5.42 ± 0.13 a | 5.74 ± 0.12 a | 5.57 ± 0.35 a | 5.37 ± 0.31 a | 5.31 ± 0.39 a | 5.53 ± 0.36 a | 2.47 ± 0.14 e | 3.27 ± 0.13 c | 3.84 ± 0.20 b | 3.06 ± 0.23 cd | 2.68 ± 0.19 de | 3.33 ± 0.20 c |
| TAA (mg/g DW) | 264.80 ± 1.34 e | 276.32 ± 1.51 c | 265.46 ± 0.28 e | 274.39 ± 0.47 d | 309.81 ± 0.38 a | 300.15 ± 1.86 b | 200.20 ± 0.56 i | 234.92 ± 1.03 h | 246.77 ± 0.97 g | 260.07 ± 0.43 f | 276.74 ± 0.56 c | 278.06 ± 1.19 c |
| EAA (mg/g DW) | 103.88 ± 0.35 f | 108.59 ± 0.86 c | 103.62 ± 0.40 f | 105.61 ± 0.06 e | 120.12 ± 0.61 a | 115.74 ± 0.09 b | 76.02 ± 0.43 j | 88.84 ± 0.30 i | 95.84 ± 0.28 h | 99.41 ± 0.74 g | 106.53 ± 0.41 d | 108.18 ± 0.55 c |
| NEAA (mg/g DW) | 160.92 ± 1.17 e | 167.73 ± 0.90 d | 161.85 ± 0.68 e | 168.78 ± 0.45 cd | 189.68 ± 0.53 a | 184.40 ± 1.78 b | 124.18 ± 0.49 h | 146.08 ± 0.74 g | 150.93 ± 0.71 f | 160.66 ± 1.08 e | 170.20 ± 0.16 c | 169.88 ± 1.05 c |
| EAA/TAA (%) | 39.23 ± 0.16 a | 39.30 ± 0.17 a | 39.03 ± 0.19 ab | 38.49 ± 0.06 de | 38.77 ± 0.18 bcd | 38.56 ± 0.21 cd | 37.97 ± 0.18 fg | 37.82 ± 0.05 g | 38.84 ± 0.05 bc | 38.23 ± 0.33 ef | 38.50 ± 0.07 de | 38.91 ± 0.19 b |
| EAA/NEAA (%) | 64.56 ± 0.43 a | 64.74 ± 0.46 a | 64.02 ± 0.52 ab | 62.57 ± 0.16 de | 63.33 ± 0.47 bcd | 62.77 ± 0.57 cd | 61.22 ± 0.47 fg | 60.82 ± 0.14 g | 63.50 ± 0.14 bc | 61.88 ± 0.86 ef | 62.59 ± 0.18 de | 63.68 ± 0.51 b |
| BCAA (mg/g DW) | 45.01 ± 0.57 f | 46.94 ± 0.42 cd | 44.68 ± 0.50 f | 45.76 ± 0.16 e | 52.88 ± 0.69 a | 50.90 ± 0.55 b | 33.16 ± 0.33 j | 38.08 ± 0.28 i | 41.52 ± 0.26 h | 43.58 ± 0.65 g | 46.69 ± 0.29 d | 47.57 ± 0.20 c |
| SAA (mg/g DW) | 6.50 ± 0.05 ef | 6.74 ± 0.15 cd | 6.43 ± 0.07 f | 6.59 ± 0.08 de | 7.77 ± 0.10 a | 7.20 ± 0.10 b | 4.67 ± 0.05 j | 5.39 ± 0.03 i | 5.76 ± 0.06 h | 6.34 ± 0.08 fg | 6.84 ± 0.14 c | 6.26 ± 0.07 g |
| AAA (mg/g DW) | 24.54 ± 0.08 g | 26.48 ± 0.16 c | 24.97 ± 0.14 f | 25.35 ± 0.23 e | 30.05 ± 0.18 a | 28.46 ± 0.10 b | 17.73 ± 0.12 j | 20.44 ± 0.09 i | 22.39 ± 0.29 h | 24.45 ± 0.26 g | 26.69 ± 0.30 c | 26.06 ± 0.05 d |
| TAA/protein (%) | 87.49 ± 4.00 a | 83.41 ± 0.32 abc | 81.76 ± 5.18 bc | 75.02 ± 2.41 e | 80.61 ± 1.38 bcd | 84.55 ± 1.42 ab | 67.86 ± 0.75 f | 75.70 ± 3.02 de | 82.20 ± 4.01 abc | 84.17 ± 1.86 ab | 78.09 ± 1.78 cde | 84.83 ± 3.96 ab |
| FAO-based EAAI | 131.80 ± 6.50 a | 126.98 ± 0.81 ab | 122.86 ± 8.06 bc | 109.64 ± 3.36 d | 118.23 ± 2.54 c | 123.48 ± 1.85 bc | 96.07 ± 0.84 e | 108.37 ± 4.60 d | 121.54 ± 5.69 bc | 118.60 ± 2.89 c | 109.39 ± 3.11 d | 121.31 ± 4.81 bc |

Values are presented as mean ± SD (n=3) on a dry-weight (DW) basis. TAA, total hydrolyzed amino acids; EAA, essential amino acids; NEAA, non-essential amino acids; BCAA, branched-chain amino acids, calculated as the sum of Val, Ile, and Leu; SAA, sulfur-containing amino acids, calculated as the sum of Met and Cys; AAA, aromatic amino acids, calculated as the sum of Phe and Tyr; EAAI, essential amino acid index. EAA comprised His, Thr, Val, Met, Ile, Leu, Phe, Lys, and Trp. EAAI was calculated using the FAO amino acid scoring pattern for individuals aged ≥ 3 years. Different lowercase letters within the same tissue and row indicate significant differences among supplementation levels (p < 0.05).

Table S2 Protein-normalized amino acid scores and essential amino acid indices of cap and stipe samples of Morchella sextelata under different levels of additional soybean meal.

| AAS item | FAO reference (mg/g protein) | Cap | | | | | | Stipe | | | | | |
| --- | --- | --- | --- | --- | --- | --- | --- | --- | --- | --- | --- | --- | --- |
|  |  | CK | 1% | 2% | 4% | 8% | 10% | CK | 1% | 2% | 4% | 8% | 10% |
| His | 16.00 | 93.71 ± 4.16 | 95.28 ± 1.35 | 84.34 ± 4.96 | 75.89 ± 1.54 | 84.20 ± 1.28 | 83.91 ± 0.43 | 73.63 ± 1.36 | 87.73 ± 5.44 | 96.38 ± 5.18 | 87.37 ± 2.76 | 85.42 ± 1.53 | 89.93 ± 5.15 |
| Ile | 30.00 | 116.63 ± 6.12 | 111.90 ± 1.22 | 109.16 ± 6.65 | 94.15 ± 3.85 | 105.30 ± 1.82 | 111.13 ± 0.84 | 87.21 ± 0.95 | 97.03 ± 2.15 | 110.56 ± 5.27 | 107.03 ± 2.07 | 101.30 ± 2.16 | 115.61 ± 4.32 |
| Leu | 61.00 | 123.69 ± 6.99 | 117.54 ± 1.36 | 112.61 ± 5.43 | 107.20 ± 1.81 | 117.79 ± 3.10 | 121.09 ± 2.52 | 91.64 ± 1.50 | 98.80 ± 4.11 | 111.60 ± 5.86 | 118.40 ± 2.67 | 110.18 ± 3.41 | 118.07 ± 5.20 |
| Lys | 48.00 | 105.55 ± 5.82 | 97.41 ± 2.24 | 97.25 ± 6.36 | 88.53 ± 2.13 | 88.15 ± 0.61 | 99.07 ± 1.49 | 84.94 ± 0.52 | 99.62 ± 4.09 | 105.54 ± 5.41 | 106.78 ± 2.58 | 101.14 ± 2.64 | 109.37 ± 4.98 |
| Met+Cys | 23.00 | 93.35 ± 5.07 | 88.42 ± 1.85 | 86.12 ± 5.82 | 78.34 ± 1.59 | 87.89 ± 1.31 | 88.18 ± 1.76 | 68.81 ± 1.27 | 75.49 ± 3.74 | 83.44 ± 4.85 | 89.26 ± 0.70 | 83.90 ± 3.04 | 83.03 ± 4.82 |
| Phe+Tyr | 41.00 | 197.77 ± 9.39 | 194.98 ± 1.64 | 187.57 ± 11.80 | 169.05 ± 6.44 | 190.70 ± 3.54 | 195.55 ± 1.96 | 146.60 ± 2.89 | 160.65 ± 7.00 | 181.98 ± 10.35 | 192.94 ± 2.59 | 183.69 ± 5.18 | 193.90 ± 8.78 |
| Thr | 25.00 | 175.71 ± 10.73 | 170.66 ± 2.03 | 166.81 ± 11.38 | 146.07 ± 6.03 | 163.95 ± 4.75 | 162.46 ± 1.21 | 144.08 ± 0.77 | 152.45 ± 4.37 | 169.60 ± 9.77 | 165.93 ± 1.77 | 154.42 ± 1.90 | 178.89 ± 6.99 |
| Trp | 6.60 | 271.01 ± 11.24 | 262.66 ± 4.74 | 260.43 ± 29.16 | 222.25 ± 14.66 | 209.47 ± 17.36 | 235.92 ± 17.84 | 126.76 ± 7.19 | 159.86 ± 10.50 | 193.44 ± 1.47 | 150.16 ± 14.10 | 114.79 ± 10.58 | 153.49 ± 3.10 |
| Val | 40.00 | 95.72 ± 5.04 | 91.02 ± 0.78 | 90.32 ± 5.44 | 78.68 ± 3.55 | 85.39 ± 2.11 | 90.46 ± 1.58 | 75.84 ± 0.90 | 83.33 ± 3.31 | 92.70 ± 4.88 | 91.71 ± 2.21 | 85.33 ± 1.28 | 96.00 ± 5.03 |

Values are presented as mean ± SD (n=3). Amino acid contents were first expressed as mg g⁻¹ protein, after which amino acid scores (AAS) were calculated using the FAO amino acid scoring pattern for individuals aged ≥ 3 years. AAS was calculated as follows: AAS = amino acid content in the sample protein (mg g⁻¹ protein)/corresponding FAO reference value (mg g⁻¹ protein) × 100. Met+Cys and Phe+Tyr were evaluated as the sulfur-containing and aromatic amino acid combinations, respectively. An AAS of 100 denotes equivalence to the corresponding reference value.

Table S3 Primer sequences used for qRT-PCR validation.

| Gene | F (5’-3’) | R (5’-3’) |
| --- | --- | --- |
| Act | GTACCCTGGTATTGCCGACC | GGACGATGGAAGGACCACTC |
| K01792 | GGCCTGATCTACTCGGTCAC | TGAAGTCGAACGTCTCGTTG |
| QARS | GTTTCCTTGCAGCTCTCCAC | TTTGGAGTGGCCAATGTGTA |
| H6S33_003786 | TTTGAGCTCCGTGAAGTCCT | AGCAAATTTCACCACCTTGG |
| H6S33_009196 | TCACTGCTGCGGATTACAAG | TCGAACGGCTGTTTCTCTTT |
| H6S33_010299 | GTAGCACCGATCTCGAGGAG | GAGTCATGTGTGCGGTATGG |
| KARS | TGATAACGCTCCTGTTGCTG | CGTGAGTTCCTCCTCCTCAG |
| PDC | CGGTGTTGGTGAATTGTCTG | TGGGGACGTGTTCACTGTAA |
| GDH2 | ACCGTGTTCTTGTGGAGGAC | GGTGCCGTTTGTAACGATCT |
| pfkA | AGCCACCAGTTGTAGGATGG | ATACGGGTGTCGAGTCCAAG |
| H6S33 005901 | GGAGACCACCGTCACAAAGT | GCTCCTTCTCACCAAACAGC |
| H6S33_000923 | CGGAATCAAGATCTCCGGTA | GTAGCGGGACATCCACAAGT |
| H6S33 009631 | GGTGTCTGCATGCTCTTCAA | AATACGTTCGCACCACCTTC |

Table S4 Summary of RNA-sequencing data quality and reference-genome mapping statistics.

| Sample Name | Raw reads | Clean reads | Q20(%) | Q30(%) | GC (%) | Mapping ratio(%) |
| --- | --- | --- | --- | --- | --- | --- |
| CKC_1 | 45588820 | 45283214 | 99.12 | 95.7 | 50.11 | 93.24 |
| CKC_2 | 39492752 | 39176354 | 99.12 | 95.88 | 50.09 | 93.28 |
| CKC_3 | 45323190 | 44937728 | 99.11 | 95.82 | 50.02 | 93.46 |
| HC_1 | 43626724 | 43230236 | 99.08 | 95.76 | 50.16 | 92.6 |
| HC_2 | 40882748 | 40511576 | 99.09 | 95.74 | 50.11 | 92.72 |
| HC_3 | 46932848 | 46502948 | 99.1 | 95.84 | 50.03 | 92.91 |
| LC_1 | 46832872 | 46399536 | 99.06 | 95.72 | 49.86 | 89.26 |
| LC_2 | 45441938 | 44998796 | 99.06 | 95.72 | 49.85 | 89.41 |
| LC_3 | 47135300 | 46721118 | 99.09 | 95.86 | 49.81 | 88.98 |
| MC_1 | 46042178 | 45631646 | 99.09 | 95.75 | 50.32 | 89.78 |
| MC_2 | 43697904 | 43320542 | 99.09 | 95.76 | 50.41 | 89.85 |
| MC_3 | 47849228 | 47436822 | 99.1 | 95.8 | 50.24 | 89.74 |
| CKS_1 | 43985974 | 43626302 | 99.08 | 95.7 | 49.73 | 94.97 |
| CKS_2 | 46799574 | 46351166 | 99.07 | 95.75 | 49.8 | 94.86 |
| CKS_3 | 47176216 | 46808486 | 99.11 | 95.9 | 49.81 | 95 |
| HS_1 | 46136212 | 45790188 | 99.12 | 95.89 | 49.95 | 93.88 |
| HS_2 | 40430198 | 40121728 | 99.1 | 95.74 | 49.86 | 93.69 |
| HS_3 | 43338854 | 42971426 | 99.08 | 95.7 | 49.84 | 93.52 |
| LS_1 | 45928144 | 45573994 | 99.13 | 95.9 | 49.89 | 93.43 |
| LS_2 | 43796766 | 43430772 | 99.11 | 95.86 | 49.92 | 93.5 |
| LS_3 | 43725312 | 43339536 | 99.1 | 95.77 | 50.01 | 93.3 |
| MS_1 | 44289962 | 43902538 | 99.04 | 95.5 | 49.79 | 91.84 |
| MS_2 | 43594280 | 43195338 | 99.06 | 95.64 | 49.7 | 92.29 |
| MS_3 | 42993956 | 42585052 | 99.08 | 95.79 | 49.72 | 91.9 |

Fig. S1 qRT-PCR validation of selected differentially expressed genes in the cap and stipe of Morchella sextelata. (A) Expression of six selected genes in the cap. (B) Expression of six selected genes in the stipe. The left and right y-axes represent TPM values and qRT-PCR fold changes 2^^−ΔΔCt^, respectively. Data are presented as mean ± SD of three biological replicates. Asterisks indicate significant differences between treatments connected by horizontal lines (p < 0.05).
